# Supplementary material for: Are carbon emissions trading and green financial instruments synergistic? -Comprehensive quantitative research based on content analysis
Source: PLoS One. 2024 Mar 7;19(3):e0298601. doi: 10.1371/journal.pone.0298601 (PMC10919601; doi:10.1371/journal.pone.0298601)
Supplement: S1 Data — (ZIP) [file pone.0298601.s001.zip › Data/地级市Z_F工作报告.zip!/╣π╬≈/║╪╓▌╩╨/└┤╫╘╣1⁄2╓┌║┼í╛┬φ┐╦╩2╛▌═°í┐_2017.docx]

一、2016年工作回顾

2016年,在自治区党委、政府和市委的正确领导下,在市人大、政协的监督支持下,我们紧紧围绕市三届人大七次会议、四届人大一次会议确定的目标任务,解放思想,迎难而上,用心做事,精准施策,统筹推进经济社会发展,实现了“十三五”良好开局。

——经济发展实现新突破。经济呈现总体平稳、稳中有进的发展态势。撤地设市以来,全市地区生产总值首次突破500亿元大关,预计达520亿元,增长8.5%,进入全区经济增长“第一方阵”；财政收入首次突破50亿元大关,达到50.9亿元,增长8%,高于全区平均增幅2.8个百分点。

——特色产业获得新提升。获授权组建全国碳酸钙专委会,荣获“中国贺州·重钙之都”称号,成为广西建筑产业现代化综合试点城市,碳酸钙产值突破150亿元,增长28.5%。生态健康产业蓬勃发展,获评为“世界长寿市”和全国第一个县域全覆盖的“中国长寿之乡”,荣获“中国长寿美食之都”称号,入选“十三五”国家服务业综合改革试点市、国家产城融合示范区建设单位、首批国家级医养结合试点市,及国家第二批全域旅游示范区创建名单。

——城乡发展呈现新景象。北堤公园、大钟山公园初步建成开园,结束了建市以来城区没有公园的历史。市文化中心成为城区新地标,第十届广西(贺州)园林园艺博览会项目建设加快推进,贺州广场提升改造等为民办实事项目基本完成,城区交通畅通、路灯亮化提升等市政工程深入实施,城市功能品质和公共服务水平进一步提升。全面推进“生态乡村”建设,新建农村新型生态社区150个,贺街入选中国第一批特色小镇和第四批美丽宜居小镇示范名单,3个村入选全国美丽宜居村庄示范名单,18个村列入第四批中国传统村落名录,农村人居环境进一步改善。

——脱贫攻坚取得新成效。精准扶贫工作全面推进,“9.1万贫困人口脱贫销号、全市58个贫困村脱贫出列、八步区脱贫摘帽”三项工作通过自治区核验,顺利完成自治区下达的年度任务。城镇居民人均可支配收入、农民人均纯收入分别增7.5%和10%。

——县域经济展现新亮点。八步区、平桂区保持良好发展势头,地区生产总值、规上工业总产值增速均高于全市平均水平；平桂区正式挂牌成立,形成发展新格局；八步区获评2016年度国家有机产品示范创建区；钟山农业农村工作获得自治区肯定,广西中小城市建设试点工作成效初显；昭平获评为中国百佳深呼吸城市、“中国长寿美食之乡”和“中国有机茶之乡”；富川上榜国家农产品质量安全县名单,被国家纳入重点生态功能区,获评为全国电子商务进农村示范县和全国计划生育优质服务先进县。

一年来,我们主要做了以下工作:

一是精准施策稳增长,经济基本面保持稳定。

二是深化改革提效能,发展活力持续释放。

三是向东开放增活力,发展空间进一步拓宽。

四是绿色发展优生态,人居环境更加宜居。

五是推动共享惠民生,群众获得感不断增强。

二、2017年工作安排

今年是党的十九大召开之年,是实施“十三五”规划、深化供给侧结构性改革的关键一年。

2017年政府工作的总体要求是:

全面贯彻党的十八大和十八届三中、四中、五中、六中全会及中央、自治区经济工作会议精神,以习近平总书记系列重要讲话精神和治国理政新理念新思想新战略为引领,按照“五位一体”和“四个全面”战略布局要求,围绕自治区第十一次党代会“营造‘三大生态’,实现‘两个建成’”的奋斗目标和市第四次党代会决策部署,坚持稳中求进工作总基调,积极适应把握经济发展新常态,深入推进供给侧结构性改革,加快实施“五大战略”和“五大攻坚行动”,全力以赴稳增长调结构,坚定不移抓改革促开放,发展壮大碳酸钙、新型建筑材料和以旅游文化养生产业为主抓手的生态健康产业等“三大千亿元产业”,加快补齐基础设施和民生短板,促进经济持续平稳健康发展和社会和谐稳定,以优异成绩迎接党的十九大胜利召开。

根据上述总体要求,初步考虑经济社会发展主要预期目标是:地区生产总值增长7.5%,财政收入增长6.5%,规模以上工业增加值增长5%,固定资产投资增长10%,社会消费品零售总额增长11%,进出口总额增长10%,城镇居民人均可支配收入和农民人均纯收入分别增长9%和10%,居民消费价格涨幅控制在3%以内,城镇登记失业率控制在4.2%以内,常住人口城镇化率达46%,节能减排控制在自治区下达目标内。

为实现上述目标,我们将重点抓好以下六个方面工作:

(一)全力推进精准脱贫攻坚。确保完成年度“7.17万贫困人口脱贫销号,全市93个贫困村脱贫出列,富川脱贫摘帽”任务。

推进重点领域攻坚。统筹资金约8亿元,重点用于贫困村基础设施建设,改善群众生产生活条件。抓好产业扶贫,完善财政扶贫贴息资金和风险补偿等政策,实现扶贫产业项目在贫困村“全覆盖”。尤其要畅通“IPO”绿色通道,吸引资本市场助力脱贫攻坚。

强化后续发展能力建设。扎实推进教育扶贫工作,组织实施教育结对、教育精准资助、职业教育扶贫攻坚“三大工程”,确保建档立卡贫困家庭子女初中毕业后100%升学就读普通高中或中职学校,中职中专学段建档立卡在校生100%就业创业或升学深造。实施健康扶贫工程,完善大病保险、医疗救助、商业健康保险等制度衔接机制力争实现对2017年预脱贫的7.17万贫困人口全覆盖。

以良好作风促脱贫保实效。坚持把脱贫摘帽作为贫困县区党政领导班子、领导干部评优评先、提拔使用的重要依据,出台《贺州市乡镇领导班子和党政主要领导抓脱贫攻坚政绩考核办法》,层层压实市、县、乡三级领导集中力量抓脱贫攻坚责任。严格落实扶贫考核机制、督查巡查机制、第三方评估机制,进一步完善对乡镇党政领导班子、专责小组、定点扶贫单位的脱贫攻坚工作绩效考评,倒逼工作责任落实。

(二)做大做强实体经济。以推进供给侧结构性改革为主线,全面落实“三去一降一补”改革任务,促进产业转型升级。

推动工业经济提质增效。加大工业项目投资力度,力争完成工业投资305亿元以上。大力发展碳酸钙和新型建筑材料产业集群,加快推进力丰实业等项目建设,推动冠成新材料等项目竣工投产,同时大力清理规模小、污染大的“作坊式”企业,着力打造布局合理、关联度高、互补性强的碳酸钙产业集群；加快发展新型墙体、钢构件等新型建筑材料,推进超超新材等项目建设,培育壮大以装配式建筑为主导的新型建筑材料产业集群。

大力发展“长寿·养生”经济。以发展旅游文化养生产业为主抓手,加快国家“医养结合”试点单位和全域旅游示范区建设,培育壮大生态健康产业“四个新业态”。抓紧推进“四个旅游小镇”建设,加快建设特色县域旅游区,完善全域旅游发展布局。扎实推进“梦里黄姚”创意城等文化旅游项目,积极创建姑婆山国家级旅游度假区,力争黄姚古镇、姑婆山景区成功创建“5A”级景区。

加快发展现代特色农业。落实粮食安全责任制,确保粮食播种面积稳定在199万亩、总产量稳定在70.5万吨左右。加快推进农业供给侧改革,实施现代特色农业产业品种品质品牌“10+3”提升行动,大力发展有机循环、富硒和休闲农业,积极创建“广西农业标准化示范市”。继续推进现代特色农业示范区建设,力争实现获认证的自治区级示范区县域全覆盖。

培育壮大现代服务业。扎实推进“十三五”国家服务业综合改革试点市建设。培育壮大生产性服务业,探索搭建中小企业征信平台,推进普惠金融建设；培育壮大和规范发展现代物流业,启动贺州物流公共信息服务平台前期工作,力争开工建设贺州农产品物流园、快递物流园,积极创建国家产城融合示范区；加快建设现代服务业集聚区,抓好石材及碳酸钙新材料交易中心等自治区级集聚区建设,实施高新区产学研基地、八步古柏汽车城等现代服务业项目,推进市生态科技服务集聚区建设。

精准帮扶企业。贯彻落实中央、自治区系列降成本政策措施,降低企业综合成本。探索推行区域电网和工业园区增量配电网建设运营模式,降低企业用电成本。继续落实并完善“营改增”试点政策。全面盘活存量资金,落实有关政策措施,降低社保费率和缴费基数,为企业减负增效。实施商标品牌战略,提升企业的市场竞争力和可持续发展能力。

推进重点领域改革。深化“放管服”改革,继续推进简政放权。推广“多规合一”试点成果应用,完善“一个平台管项目审批”制度,全面提升项目审批效率。深化财税体制改革,完善预算管理制度,持续推进预决算公开。强化预算执行管理,完成年度预算任务。推进资源税改革,配合做好烟叶税等税收立法工作。加强地方政府债务管理,确保财政可持续。深化国资国企改革,强化市场化运作,整合提升现有的四大平台公司向市场直接融资能力。

强化创新驱动发展。推进产业创新平台建设,加快国家碳酸钙产品质量监督检验中心、广西碳酸钙资源综合利用重点实验室培育基地建设,在微波技术应用、果蔬保鲜与深加工研究等领域培育若干自治区级重点实验室。加强工程技术研发平台建设,推进金源稀土与中科院等单位共同组建的国家级工程技术研究中心建设,打造一批自治区级工程技术研究中心。

(三)全力攻坚重大项目投资。着力破解瓶颈问题,掀起项目投资新高潮,持续拉动经济增长。

积极扩大有效投资。认真贯彻落实国家、自治区系列促投资政策,采取有效措施促进投资增长,力争年内完成固定资产投资725亿元以上。突出抓好重大项目建设,全速推进356项市级层面开竣工、续建和预备的重大项目,力争年内完成投资331亿元。

扎实推进基础设施建设。深入实施基础设施建设大会战,进一步补齐基础设施短板。铁路方面,继续做好贺州至梧州城际铁路、柳韶铁路贺州段等项目前期工作。公路方面,实现永贺高速主线投入使用,加快贺巴高速公路贺州段建设,新开工永贺高速贺州支线,做好信梧高速贺州段、广东连山至贺州高速贺州段前期工作,力争年内开工。

集中力量开展征地拆迁攻坚。对批而未征、征而未供、供而未用的土地进行专项整治,着力解决“两地”安置等历史遗留问题,确保全市供地率达60%以上。重点推进生态新城和江南东、西片区约1万亩土地的征收储备工作,全面盘活闲置土地。

(四)持续提升向东开放水平。牢牢把握“向东开放”战略,加快融入珠三角经济圈,构建多维开放体系。

积极融入“两带”建设。充分利用地处“珠江—西江经济带”和“粤桂黔高铁经济带”两带叠加的优势,以丰富的生态资源和历史人文景观为依托,加快建设、营销黄姚古镇等一批“看得见山水、记得住乡愁”的特色城镇和景区,深度融入高铁沿线旅游圈,提升打造“粤港澳后花园”品牌。

强化精准有效招商。以工业园区为主平台,制定鼓励企业入驻发展的特色招商引资政策,充分发挥好园区招商引资排头兵作用。大力推行产业链招商,围绕碳酸钙、新型建筑材料、生态健康三大产业集群,瞄准世界、中国、民营500强企业和行业领军企业,引进一批引领型、补链型、核心型的产业项目,以大企业促进大产业、推进大开放。

推动进出口回升向好。继续实施第二轮“加工贸易倍增计划”和“自主品牌出口增长行动计划”,提升出口商品的质量和档次。加强出口品牌建设,支持平桂飞碟进出口公司等符合条件的企业申报“广西出口名牌”。支持海关畅通报关渠道,提高报关效率,鼓励我市企业在市内报关出口,努力培育新的进出口企业。

(五)加快推进“生态贺州”建设。巩固和提升贺州的生态优势,加快绿色崛起步伐。

加强生态环境保护与建设。扎实推进富川龟石、昭平桂江、贺州合面狮湖等国家湿地公园建设。加大生态林和古树名木保护力度,扎实推进现代特色林业(核心)示范区建设,力争全市主要林业生态资源实现示范区创建全覆盖。全面完成国有林场改革,扎实推进姑婆山全国森林康养基地试点建设,争创全国森林旅游示范市。

提升城市综合品质。坚持规划先行,运用“多规合一”试点成果,完成城市总体规划修编。以承办第十届广西(贺州)园林园艺博览会和贺州建市15周年为契机,开展全面提升城市建设五年行动,扎实推进“一江两岸”、生态新城、城东新区等建设,确保园博园在今年6月份左右开园,力争启动古城项目建设。加大旧城区改造力度,扎实推进总投资8.5亿元的灵峰山以西片区旧住宅区综合整治改造工程。抓好市政基础设施建设,继续推进北堤公园二期、大钟山公园二期建设,建成爱莲湖公园等一批城市公园。

统筹推进城乡一体化进程。加大在财税改革、土地政策、产业发展等方面对县域发展的支持力度。继续推进特色重点镇建设攻坚行动,重点抓好贺街全国特色小镇建设及莲塘、公会、公安等第三批广西百镇建设示范工程。持续巩固“生态乡村”建设成果,扩大自治区级生态乡镇、生态村的覆盖面。

(六)持续保障改善民生。坚持以民为本,多办为民谋利的实事,使发展成果更多、更公平的惠及全市人民。

加强就业和社会保障体系建设。抓好高校毕业生、返乡农民工、建档立卡贫困户的就业工作。扎实推进火车站站前片区棚户区改造等项目,按自治区部署开工一批保障性住房、棚户区改造和农村危房改造项目。深入实施城乡居民收入倍增计划,缩小城乡居民收入差距。严厉打击拖欠农民工工资行为,保障农民工合法权益。推进“全民参保”行动,加强社会保险扩面和基金征缴。全面推进城乡居民基本医疗保险制度整合、基本医疗费用即时结算和异地就医结算。

抓好卫生与健康工作。加快建设市人民医院城东分院、市中医院综合大楼、钟山县中医院住院楼等项目,启动城市公立医院综合改革,完成市医养信息智慧平台系统、120指挥调度大数据指挥系统建设。进一步完善财政投入保障制度和分级诊疗制度。加强计划生育服务管理,稳步实施全面两孩政策,继续开展诚信计生示范单位和全国新一轮计划生育优质服务先进单位创建活动。抓好出生人口性别比综合治理,加大整治“两非”工作力度。深入做好公共卫生与卫生应急各项工作,建立疾病应急救助制度,继续推动新一轮防治艾滋病攻坚工程。加强医疗质量管理和医院监管,开展“平安医院”建设,继续做好妇幼健康服务。

坚持教育优先发展。筹措资金2.66亿元,用于258所学校“全面改薄”工作。启动城东片区新建幼儿园、小学项目,加快平桂第二实验小学等项目建设,实现贺州第二高中扩建、贺州职业学院实训基地等项目竣工,确保市示范性综合实践基地项目建成使用。鼓励发展多元普惠性幼儿园,扩大优质学前教育资源。推进义务教育均衡发展,确保八步、钟山通过自治区达标验收,富川通过国家达标验收。

提升公共文化和体育水平。推进市文化中心、“四馆”、村级公共服务中心及村村通广播电视无线发射台站等基础设施建设。深入开展“和谐文化在基层”系列群众文化活动,持续抓好公共文化基础设施场馆免费开放、农村文化建设专项补助项目和农村公益电影放映等文化惠民工程。积极申报国家历史文化名城,做好第九届广西音乐舞蹈比赛筹备工作。与广东主流媒体合作拍摄电视剧《外来媳妇本地郎》、制作“发现贺州”专题片,并加大在各类媒体平台的宣传推介力度,不断提升我市在珠三角地区的影响力。加强文化市场监管,深入开展“扫黄打非”进基层等工作。加大体育人才培养力度,推进市体育运动学校迁建工程、平桂文化体育中心建设,实现贫困村体育基础设施全覆盖。做好2017年广西青少年锦标赛等赛事筹备工作,积极开展群众性赛事活动,不断提高人民身体素质。

加强和创新社会治理。强化社会主义核心价值观教育,提升市民道德意识。扎实推进诚信贺州建设,支持金融部门化解、处置不良贷款,坚决打击逃废债行为。健全应急管理体制机制,提高综合防灾减灾救灾能力。落实安全生产“党政同责、一岗双责、齐抓共管、失职追责”机制,健全安全风险预防控制体系和隐患排查治理体系建设,深入开展隐患排查治理专项活动,坚决防范遏制重特大事故。坚持源头严防、过程严管、风险严控,巩固和完善食品药品安全监管体系,提升食品药品安全保障水平,积极创建广西食品安全城市。深入推进法治贺州、平安贺州建设,加快构建立体化现代化社会治安防控体系。

同时,进一步加强国防动员和“双拥”工作,充分发挥工会、共青团、妇联等人民团体和社会组织参与管理、服务的重要作用,认真落实党的民族、宗教和侨务政策,切实做好机构编制、审计、统计、工商、史志、档案、工会、二轻、供销、住房公积金、税务、检验检疫、质量技术监督、移民搬迁、应急管理、气象、防震减灾、驻梧管理等方面工作。

三、全面加强政府自身建设

以全面从严治党统领政府各项工作,依法全面履行政府职能,努力打造人民满意政府。

坚持依法行政。建立健全党政主要负责人为第一责任人、政府负总责、法制办牵头、各职能部门为主体的推进法治政府建设领导机制。加强和改进政府立法工作,科学制定政府年度立法计划,健全完善政府立法项目立项评审、向社会公开征集意见和立法听证制度,抓好城乡建设与管理、环境保护、历史文化保护等方面的立法工作。坚持依法行政,实行负面清单、权力清单、责任清单动态管理,贯彻落实中央推进城市执法体制改革,推动乡镇延伸综合行政执法,实现“四所合一”“多所合一”。深入开展“七五”普法,推进全民普法守法,营造公平公正的社会环境。

优化政府服务。加快“网上政府”建设,推行“互联网+政务服务”,力争实现各类服务事项全流程网上运行,让企业和群众办事更方便、更快捷。优化公共服务供给,推动公共服务事项进驻各级政府服务中心集中办理。全面清理涉及群众办事的各种不必要证明。加大政务公开力度,推动决策、执行、管理、服务和结果“五公开”,扩大公众参与公共政策制定、执行和监督渠道,做好重大政策解读和政务舆情回应工作。

提高决策效率。坚持依法决策、民主决策、科学决策,建立完善重大行政决策社会稳定风险评估机制,加强重大决策第三方评估,严格行政决策责任追究。构建公众参与、专家论证相结合的行政决策机制,探索完善重大决策专家咨询论证制度。坚持“三重一大”事项报告制度,坚决服从市委的领导,自觉接受人大的法律监督和政协的民主监督,虚心接受社会各界监督,认真听取意见建议,切实办好人大代表建议和政协委员提案。

强化作风建设。全面贯彻落实中央八项规定精神和国务院“约法三章”,严格控制“三公”经费,严格履行党风廉政建设“一岗双责”。巩固“两学一做”学习教育成果,重点在挖掘“人”的潜力上下功夫,促进专业办事,优化干部队伍配置,提高人岗相适度,做到“搞经济的懂经济”“搞项目的懂项目”,让更多专业的人办专业的事；鼓励专心做事,提升各级领导干部精心谋事、潜心干事、用心做事的责任感和紧迫感,形成见贤思齐的良好作风导向,推动全市上下“心往一处想、劲往一处使”,努力营造想干事、能干事、干成事的良好氛围；坚持专注成事,探索建立容错免责机制,让领导干部始终保持专注发展的政治定力,做到应该完成的任务顶着压力也要完成,应该承担的责任迎着风险也要承担,推动政府工作提速提质提效。

各位代表!新征程蕴含新希望,新蓝图昭示新辉煌。让我们紧密团结在以习近平同志为核心的党中央周围,在自治区党委、政府和市委的正确领导下,以昂扬进取的精神状态、只争朝夕的豪迈干劲、众志成城的强大合力,不忘初心,继续前行,主动作为,不断开创贺州改革发展新局面,以优异成绩迎接党的十九大胜利召开!
